# Why Huddle? Ecological Drivers of Chick Aggregations in Gentoo Penguins, *Pygoscelis papua*, across Latitudes

**DOI:** 10.1371/journal.pone.0145676

**Published:** 2016-02-03

**Authors:** Caitlin Black, Ben Collen, Daniel Johnston, Tom Hart

**Affiliations:** 1 University of Oxford, Department of Zoology, Oxford, United Kingdom; 2 University College London, Centre for Biodiversity & Environment Research, London, United Kingdom; 3 British Antarctic Survey, King Edward Point Research Station, Cumberland East Bay, South Georgia; Phillip Island Nature Parks, AUSTRALIA

## Abstract

Aggregations of young animals are common in a range of endothermic and ectothermic species, yet the adaptive behavior may depend on social circumstance and local conditions. In penguins, many species form aggregations (aka. crèches) for a variety of purposes, whilst others have never been observed exhibiting this behavior. Those that do form aggregations do so for three known benefits: 1) reduced thermoregulatory requirements, 2) avoidance of unrelated-adult aggression, and 3) lower predation risk. In gentoo penguins, *Pygoscelis papua*, chick aggregations are known to form during the post-guard period, yet the cause of these aggregations is poorly understood. Here, for the first time, we study aggregation behavior in gentoo penguins, examining four study sites along a latitudinal gradient using time-lapse cameras to examine the adaptive benefit of aggregations to chicks. Our results support the idea that aggregations of gentoo chicks decrease an individual’s energetic expenditure when wet, cold conditions are present. However, we found significant differences in aggregation behavior between the lowest latitude site, Maiviken, South Georgia, and two of the higher latitude sites on the Antarctic Peninsula, suggesting this behavior may be colony specific. We provide strong evidence that more chicks aggregate and a larger number of aggregations occur on South Georgia, while the opposite occurs at Petermann Island in Antarctica. Future studies should evaluate multiple seabird colonies within one species before generalizing behaviors based on one location, and past studies may need to be re-evaluated to determine whether chick aggregation and other behaviors are in fact exhibited species-wide.

## Introduction

Aggregations of young animals are common in a range of endothermic and ectothermic species, yet the behavior’s adaptive purposes depend on a species’ social behaviors and local conditions. In avian species, crèching or aggregations of chicks may stem from ecological, morphological, or social adaptations [1]. Ecologically, adverse weather conditions and lower than average ambient temperatures can cause chicks to aggregate more frequently or in more dense groups, for example in the common bushtit, *Psaltriparus minimus* [2], white pelican, *Pelecanus erythrorhynchos* [3], and house sparrow, *Passer domesticus* [4]. Additionally, morphological restrictions, including poor insulation, may cause chicks of smaller species, or those with altricial young, to aggregate [1]. Furthermore, sociality may contribute to the formation of chick aggregations in birds of the same or, rarely, different species (tree swallow, *Iridoprocne bicolour*, barn swallow, *Hirundo rustica*, and cliff swallow, *Petrochelidon pyrrhonota* [5]), although little research exists on this topic. Of all the bird families, penguins (*Spheniscidae*) are well known to exhibit aggregation behaviors during both the winter and summer months [6], and the purpose of aggregations is highly dependent on the species.

Many species of penguins form aggregations (e.g. emperor, *Aptenodytes forsteri* [7]; king, *A*. *patagonicus* [8, 9]; chinstrap, *Pygoscelis antarcticu*s [10, 11]; Adélie, *P*. *adeliae* [12–14]; African, *Spheniscus demersus* [15, 16]), while others have never been observed exhibiting this behavior (e.g. little, *Eudyptula minor*, yellow-eyed *Megadyptes antipodes*, Magellanic, *Spheniscus magellanicus*, Galapagos, *Spheniscus mendiculus*, and Humboldt penguins, *S*. *humboldti* [6]). Those that do form aggregations (formally called ‘créches’; see [6] for nomenclature) do so for three known benefits: 1) reduced thermoregulatory requirements, 2) avoidance of aggression from unrelated adults and 3) lower predation risk. Firstly, reduced energy requirements for thermoregulation may serve as the primary function of chick aggregations: in other words, that which served as the first evolutionary adaptation [6]. In king and Adélie penguins, aggregations have increased either in size and compactness [9] or have only occurred when colder, harsher weather conditions are present [14]; while in other species, such as the African penguin, aggregations have been observed even during warm temperature conditions [16]. Therefore the necessity of forming aggregations for thermoregulatory purposes is dependent on the species and potentially the specific colony, although, to our knowledge, this has not yet been studied.

Secondly, chicks may form aggregations as a result of aggression from unrelated adults, where chicks are often corralled into one location by “floater” or nesting adults [16, 17]. Chicks in aggregations are less disturbed by adults than individuals outside of aggregations [16]. This behavior is exhibited by chinstrap, rockhopper, Adélie, and African penguin species [10, 12, 16–18] and serves as another explanation of chick aggregations.

Thirdly, in a classic example of both the group vigilance hypothesis and the predator dilution effect, chick aggregations may reduce predation risk by increasing the detection of predators while decreasing the chance of individual predation [19]. In Adélie penguins, fewer chicks are killed by predators when aggregating in large numbers compared with those in smaller aggregations [13], while in other species (e.g. Adélie penguins, Rockhopper penguins, chinstrap penguins), predation events only occur when individuals are not part of an aggregation [10, 13, 18]. There also may be an unexplained social benefit to aggregating as a chick, although no studies have yet provided evidence of sociality as a potential driver of aggregation.

In gentoo penguins, *Pygoscelis papua*, chick aggregations are known to form during the post-guard period, yet the cause of these aggregations is poorly understood [6]. As a species which has a large spatial range, colonizing sub-Antarctic islands and areas along the Antarctic Peninsula, it is possible that chick aggregation behavior may stem from any of the three main drivers–thermoregulation, predation, or adult aggression–for different adaptive purposes. In addition, the principal driver may vary latitudinally with colony location. Because gentoo penguins are well known for their asynchronous breeding, dependent on the colony location [20], and the growth rate within colonies and post-guard period varies depending on breeding site [21], there will likely be large differences in the timing of the post-guard phase between colonies and therefore the timing of aggregations. Here, for the first time, we examine the adaptive benefit of chick aggregation behavior in gentoo penguins, using time-lapse cameras to measure the behavior. To the best of our knowledge, we are the first to compare any chick aggregation behavior at more than one colony, enabling us to examine whether this behavior is generalizable within a species. To understand this behavior in gentoo chicks, we formulated *a priori* hypotheses based on the three previously observed mechanisms behind chick aggregation behavior in other penguin species:

1. If gentoo chicks form aggregations to reduce thermoregulatory need, chicks will aggregate more 1) during cold, wet conditions, 2) when chicks are younger in age, and 3) the number of chick aggregations will change along a latitudinal gradient; colonies located farther north will form fewer aggregations due to decreased thermoregulatory output, while those located farther south will aggregate more often during harsher, colder conditions.
2. If chicks form aggregations due to aggressive advances from unrelated adults, then aggregations will occur more often 1) when more adults are present, and the adult to chick ratio is therefore higher, and 2) when fewer chicks are guarded by their parents.
3. Younger chicks will form larger aggregations and a higher proportion of chicks will aggregate when predators are present, as a means of predator avoidance.

## Methods

### Study sites

Work on South Georgia was permitted by the Government of South Georgia and the South Sandwich Islands (GSGSSI) Permits for Antarctica were issued by the UK Foreign and Commonwealth Office under the Antarctic Treaty system. Each of these permits was issued following independent ethical review of the sampling and the study received ethical approval from the University of Oxford and the Zoological Society of London.

**Fig 1:**
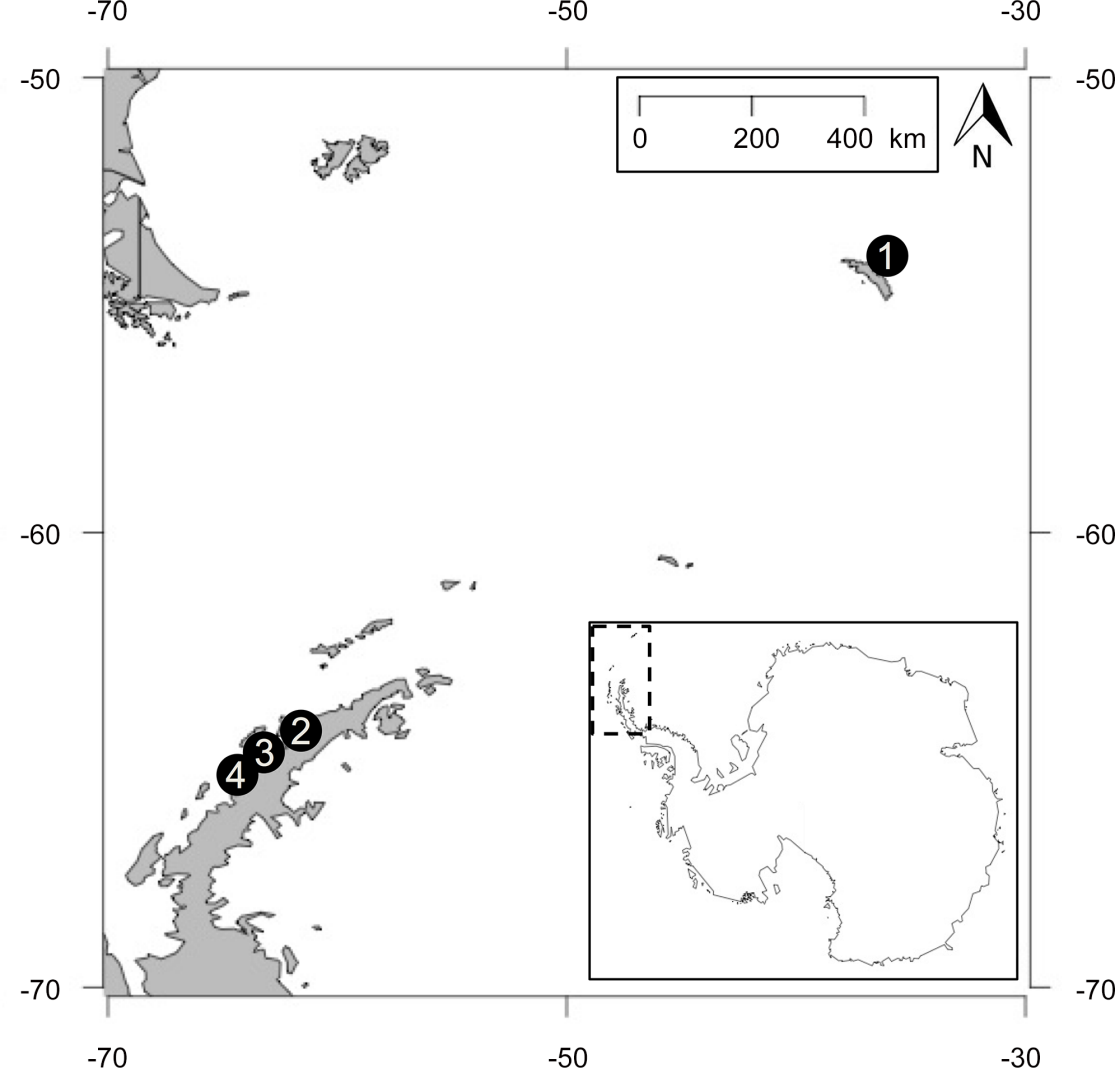
Map of studied gentoo penguin colony locations along a latitudinal gradient ranging from 1) Maiviken, South Georgia to 2) Georges Point, 3) Port Lockroy, and 4) Petermann Island on the Antarctic Peninsula.

We deployed cameras at four study sites to study chick aggregations in gentoo penguins based on a latitudinal gradient: 1) Maiviken (-36.506, -54.246) on South Georgia, 2) Georges Point (-62.670, -64.669) on Ronge Island along the Western Antarctic Peninsula (WAP), 3) Port Lockroy (-63.484, -64.823) also along the WAP, and 4) Petermann Island (-64.142, -65.172,) along the WAP (Fig 1). Petermann Island represents the southern breeding limit for gentoo penguins, located less than 11 km from the southernmost colony (Cape Tuxen [22–24]). Maiviken serves as a representation of gentoo penguins towards the more northern edge of their range (northern range ends on Crozet Island at a longitude of -45.83° [25]) and is infrequently studied. Port Lockroy, in the well-studied Palmer Archipelago, consists of a gentoo colony, which is heavily visited by tourists and has thus been studied to define tourism related impacts on the species [26]. The Georges Point colony has been studied in the past [27–29]; however, the phenology of the colony is not well defined.

Considering our three hypotheses, we must note that human visitation may affect our third hypothesis that a higher proportion of chicks will aggregate when predators are present, as a means of predator avoidance. At highly visited tourist sites (specifically Port Lockroy and Petermann Island), gentoos are habituated to human presence [26], which may influence behaviors, including the likelihood of forming aggregations. In particular, gentoos at sites frequently visited by tourists may respond differently to predators than those at sites rarely visited, due to their acclimation to humans, which in turn may influence aggregation behavior. However, the sub-colonies studied, including that at Port Lockroy and Petermann Island, are located away from tourist congregations and therefore less disturbed than other sub-colonies at the same sites, although gentoo chicks are mobile during the post-guard phase.

### Camera system

One camera was deployed at each of our four gentoo colony study sites; each camera was installed roughly 10 meters from the perimeter of nesting sub-colonies. Cameras were installed using techniques similar to those described by [30], with minor adjustments to the camera system. At each site, a Reconyx HC500 Hyperfire trail camera (Reconyx, Inc., Holmen, WI, USA) was mounted to a scaffold pole and anchored using a rock basket. The cameras were programmed in time-lapse mode to take nine photographs daily, beginning at 9:00 and ending at 17:00 (GMT-2). Each camera captured images of 14–49 nests for the full 2012–2013 breeding season (September 1- March 31, S1 Fig).

### Annotations

**Fig 2:**
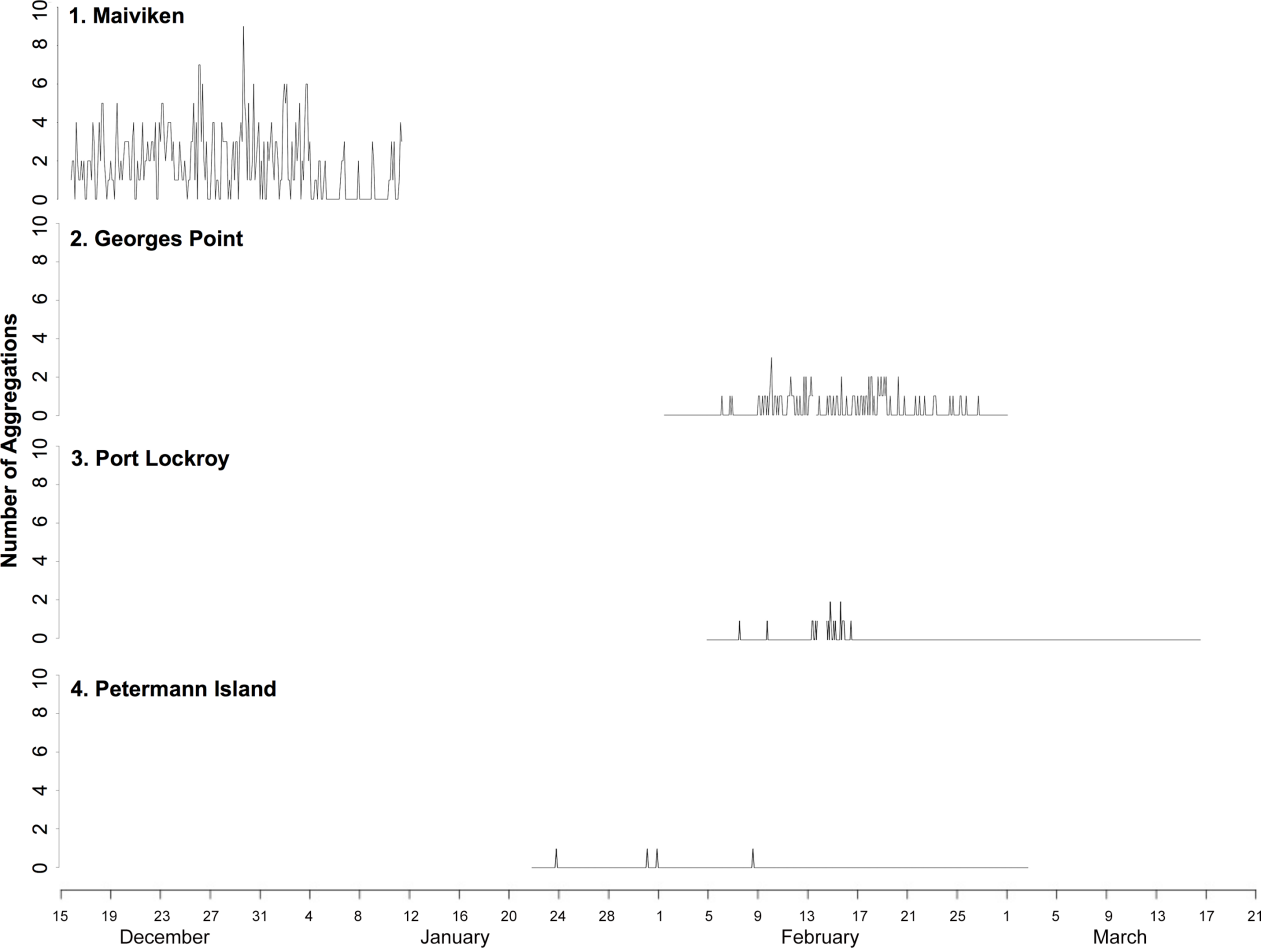
Time series demonstrating difference in the timing of the post-guard period and the number of aggregations over time at four sites: 1) Maiviken, 2) Georges Point, 3) Port Lockroy, and 4) Petermann Island.

Overall, the study period was defined as the post-guard period within a chick’s annual cycle. Each site differed in the timing of this period (Fig 2), therefore, the commencement of the post-guard period was defined as the first image in which a chick was seen unguarded (see *Guarded and unguarded chicks* in Methods), and the period was defined as terminated when either all chicks had molted to their fledgling feathers or, in cases where chicks departed the colony before the end of moult, all chicks had left the sub-colony site in view. Specifically, in the 2012–2013 breeding season, the post-guard period occurred during the following dates: Maiviken (December 16- January 12), 2) Georges Point (February 1- March 1), 3) Port Lockroy (February 5- March 17), and 4) Petermann Island (January 22- March 3; S1 Table).

### Image analysis

Counts of individuals were extracted from each image using software developed by [31]. In each image, a circle was centered over each visible individual to avoid counting individuals twice. The number of circles in each image was then extracted to determine counts. This process was used for counting both adults and chicks separately at each site in each image and all individuals at the edge of the image frame were included in our analysis. The adult to chick ratio was then determined for each image based on the counts of adults and chicks present within the camera frame.

### Chick aggregations

Although many definitions exist for a chick aggregation in penguins [6], we chose to use the definition used for Adélie penguins in [13]. We defined a chick aggregation as a minimum of three chicks in close association, where the distance between individuals was less than 35 cm, approximately half the distance between nests, consistent with pecking distance. For each image, the total number of aggregations and the number of chicks in each aggregation was determined. Using these data we calculated: mean aggregation size, standard deviation in aggregation size, total number of chicks in aggregations, and ratio of aggregating chicks to total chicks, for each image.

### Guarded and unguarded chicks

Guarded chicks were defined as being in close association with an adult and located one flipper length (~25 cm) or less from the brood patch of the nearby adult. In addition, any chicks involved in provisioning interactions or located directly on the nest with an adult were defined as guarded (aka. attended). All other chicks were counted as unattended. From this data, a ratio of guarded chicks to unguarded chicks was determined.

### Weather conditions

Images were annotated for the presence or absence of wet conditions, defined as either snow cover, precipitation in the form of snow or rain, or conditions in which chick down feathers were noticeably wet. All weather conditions are presented here as binary data. In addition, ambient temperatures were extracted from an internal thermometer in the camera housing. Wind was not measured here, although it has been linked with aggregation behavior in king penguins [9].

### Predators present

The presence of any pinnipeds or predatory birds was noted for each image and the species was determined using an identification guide [32]. Using this data, the number of predators and scavengers was determined for each image.

### Age of chicks

Because we were unable to determine the exact age of individuals and unable to differentiate between individuals from specific nests during the post-guard phase studied, we instead used the days from the start of the post-guard period (day 1 = 1, day 2 = 2, etc.) as a method to evaluate the effect of chicks aging, at a sub-colony level, in our analysis of aggregation behavior.

### Statistical analysis

All analyses were conducted in R (3.0.3 [33]) using the *stats* and *MASS* packages [34]. To account for the known differences between the angle and view within each camera frame, which likely affect the explanatory variables, we counted the total number of nests on the first day of the post-guard period at each study site and used these nest counts as an offset term (-log(x)) in each of our models (*offset* function, *stats* package). To determine the relationship between each of our explanatory variables (Table 1) and the counts of the total number of aggregations in each image, we used a generalized linear model (GLM) with a Poisson error structure. We simplified this model by omitting insignificant coefficients, including time of day, the adult-to-chick ratio (square-root transformed), and temperature (ANOVA, p = 0.02). Ultimately, for this first model, the GLM coefficients included an interaction between location and wet conditions, the ratio of guarded to unguarded chicks (log transformed), and the day within the post-guard period (residual deviance: 232.61 on 426 degrees of freedom).

**Table 1:** Results from a negative binomial GLM on the number of gentoo penguin chicks aggregating (residual deviance: 239.29 on 421 degrees of freedom) and a Poisson GLM on the number of aggregations per nest unit (residual deviance: 232.61 on 426 degrees of freedom) for each coefficient included in the models.

Response variable	Coefficient		Estimate	SE	Z	P
**Number of chicks aggregating**	**Location**	Maiviken	4.81	0.348	13.835	<0.001**
		Georges Pt.	-4.07	8.574	-4.743	<0.001**
		Port Lockroy	-0.35	<0.001	<0.001	0.999
		Petermann Isl.	-5.06	1.460	-3.466	<0.001**
	**Location: wet**	Maiviken: wet	0.96	0.864	1.116	0.264
		Georges Pt.: wet	1.10	1.171	0.936	0.349
		Port Lockroy: wet	0.33	<0.001	<0.001	0.999
		Petermann Isl.: wet	4.92	2.097	2.344	0.019*
	Temperature: wet		-0.31	0.123	-2.523	0.012*
	Post-guard day		-0.05	0.025	-2.026	0.043*
	log(guarded chicks: unguarded chicks)		-0.61	0.116	-5.256	<0.001**
**Number of aggregations**	**Location**	Maiviken	1.83	0.176	10.377	<0.001**
		Georges Pt.	-1.71	0.462	-3.696	<0.001**
		Port Lockroy	-18.58	825.6	-0023	0.982
		Petermann Isl.	-4.60	1.016	-4.507	<0.001**
	**Location: wet**	Maiviken: wet	-0.005	0.512	-0.010	0.992
		Georges Pt.: wet	0.29	0.702	0.412	0.681
		Port Lockroy: wet	14.63	825.6	0.018	0.986
		Petermann Isl.: wet	4.97	1.503	3.303	<0.001**
	Post-guard day		-0.07	0.017	-4.354	<0.001**
	log(guarded chicks: unguarded chicks)		-0.42	0.084	-4.955	<0.001**

p *<0.05

p **<0.001.

Due to overdispersion in both Poisson and quasi-Poisson GLMs, we used a negative binomial GLM (*glm*.*nb* function, *MASS* package) to determine interactions between each of the coefficients and the counts of aggregating chicks in each image. We also simplified our negative binomial GLM by omitting the time of day and the adult-to-chick ratio (square-root transformed; ANOVA, p = 0.07). Our final model included the following coefficients: 1) interactions between location, temperature, and wet conditions, 2) the ratio of guarded to unguarded chicks (log transformed), and 3) the day within the post-guard period (residual deviance: 239.29 on 421 degrees of freedom). By examining both models with and without a correlation structure and the auto-correlation in the observed residuals (*acf* function, *stats* package), we determined that temporal autocorrelation did not occur in our data set and a correlation structure was therefore omitted from the model. We must note that our sample size for the number of predators present was small across the four sites (n = 8) and was therefore not included in either model. Lastly, to understand relationships between coefficients, we examined contrasts by reordering the levels of factors (*relevel* function, *stats* package).

## Results

We found significant relationships between location, wet conditions, the ratio of guarded to unguarded chicks, and the age of chicks and each of our response variables: 1) the number of aggregations and 2) the number of chicks aggregating (Table 1). Because nest counts were used to offset the data to account for the differences in the camera view and angle between each of our study sites, the following results indicate the relationship between each coefficient and our measures of aggregation behavior per nest unit.

When examining the counts of aggregating chicks in each image, the Maiviken (South Georgia) chicks exhibited significantly more aggregation behavior (SE = 0.35, p<0.001) than either the Georges Point (SE = 0.86, p<0.001) or Petermann Island colonies (SE = 1.46, p<0.001; Table 2). However, our model for the number of aggregations at each colony revealed that not only did chick aggregations increase significantly at Maiviken compared to Petermann Island and Georges Point (SE = 0.18, p<0.001), but the number of aggregations exhibited by Petermann Island chicks also differed significantly from Georges Point (SE = 0.46, p<0.001), with chicks at Petermann Island undergoing far fewer aggregations (SE = 1.02, p<0.001).

**Table 2:** Summary of measures of variance (range, mean, and standard deviation) in three aggregation characteristics (1) average aggregation size, 2) total number of chicks in aggregations, and 3) total number of aggregations at each of our four study sites and in total across all four sites: Maiviken, Georges Point, Port Lockroy, and Petermann Island.

Aggregation measure	Measures of variance	Site				
		Maiviken	Georges Point	Port Lockroy	Petermann Island	All sites
**Average aggregation size**	Range	3–73	3–9	3–10	3–3	3–73
	Mean	10.62	3.59	4.03	3	8.11
	*sd*	12.15	1.30	1.99	0	5.34
**Total number of chicks in aggregations**	Range	0–105	0–13	0–10	0–3	0–105
	Mean	15.81	1.02	0.13	0.03	2.88
	*sd*	19.03	2.12	0.85	0.30	10.32
**Percent of chicks aggregating**	Range	0–100	0–52.53	0–100	0–37.5	0–100
	Mean	27.62	3.99	1.28	0.16	5.92
	*sd*	27.93	8.25	8.53	2.15	16.28
**Total number of aggregations**	Range	0–9	0–3	0–2	0–1	0–9
	Mean	3.59	0.28	0.03	0.01	0.37
	*sd*	1.30	0.55	0.20	0.10	1.00

For both our measures of the number of chicks aggregating and the number of aggregations, we found negative relationships between our response variables and 1) the day within the post-guard period (SE = 0.02, p<0.04; SE = 0.02, p<0.001) and 2) the ratio of guarded chicks to unguarded chicks (SE = 0.12, p<0.001; SE = 0.08, p<0.001, Table 1). In other words, the number of chicks aggregating and the total number of aggregations increased when chicks were younger and when more chicks were left unguarded by their parents.

We also found a relationship between the environmental variables of temperature and wet conditions present and the two measures of chick aggregations, although these conditions depended on the location of the colony. The number of aggregations and counts of chicks aggregating increased at Petermann Island when wet conditions were present at the colony (SE = 2.01, p = 0.02; SE = 1.42, p<0.001, Table 1). In addition, the number of aggregations at Petermann Island was significantly different from both the Georges Point (SE = 1.51, p = 0.002) and Maiviken colonies (SE = 1.50, p<0.001). We also found a significant negative relationship between temperature and wet conditions and the counts of chicks in aggregations across sites (SE = 0.12, p = 0.01), but not between the total aggregation counts in each image. In other words, as temperature increased during wet conditions, the total counts of chicks in aggregations decreased.

## Discussion

To the best of our knowledge, we are the first to study the adaptive significance of chick aggregation behavior in gentoo penguins and the first to examine this behavior at multiple colonies, covering a large geographic range, in any penguin species. Our results support the idea that aggregations occur to decrease an individual’s energetic expenditure during wet, cold conditions. However, we found significant differences in aggregation behavior across a latitudinal gradient, particularly between the Maiviken (South Georgia) site and two of the other three sites located in closer proximity on the Antarctic Peninsula, suggesting that this behavior may be colony specific, and the result of a more complex set of interactions.

The timing of the post-guard phase varied notably between Maiviken, South Georgia and the three Antarctic Peninsula sites (Fig 2). The phenology of gentoo penguins has been shown to vary between years and locations, a relationship that is influenced by sea temperature and local resource availability [21]. Because aggregation behavior is closely linked with the post-guard phase, the timing of this behavior may be dictated by parental phenological constraints. Specifically, the timing of post-guard period may be dictated by the adult need to build up reserves prior to moult, and therefore leave chicks unguarded, while the formation of aggregations may be instead dictated by a chick’s need to survive and thermoregulate without constant guard [10]. In addition, certain colonies may be more synchronous than others [20] and asynchronicity may influence chick aggregation behavior as aggregations may be smaller and less frequent when individuals enter the post-guard period at different times.

We found a significant link between temperature, increased wet conditions, and the age of chicks and our two measures of aggregation behavior (Table 1), similar to past studies focusing on this behavior in other penguin species (eg. Adélie penguins [13, 14]; emperor penguins [7]; king penguins [9]). In particular, we found that a slight decrease in temperature when wet conditions were present correlated with an increased number of chicks aggregating (Table 1). In addition, chicks appear to aggregate earlier in the post-guard period when younger and therefore more vulnerable to cold, wet conditions. Although gentoo chicks are thought to fully self-regulate body temperature roughly 15 days after hatching [35], before the post-guard period, other evidence suggests that chicks may not be able to thermoregulate independently when wet conditions are present [36]. Our study supports harsh weather conditions potentially being as influential in chick aggregating behavior as daily temperature variation.

Although our analysis revealed that, overall, aggregations are related to temperatures and wet conditions; we also found significant differences when examining each of our aggregation measures within sites (Table 1). In particular, the number of aggregations and chicks aggregating was significantly and positively related to wet conditions at the Petermann Island, Antarctic Peninsula study site, but not at the other three sites. In addition, chicks at the two study sites located at either extreme of our latitudinal gradient, Maiviken and Petermann Island, underwent different degrees of aggregation behavior: the Maiviken chicks aggregating significantly more while the Petermann Island chicks aggregated significantly less. It is possible that colonies may form aggregations using different mechanisms, depending on their location and the conditions present during the post-guard period. However, because our analysis across sites revealed general trends, it is more likely that aggregations form due to wet, cold conditions and that the differences in weather and the timing of the post-guard period between sites is driving aggregation extent.

Unlike in other penguin species (Adélie penguins [13]; African penguins [15]; king penguins [9]) we did not find evidence to support either predator avoidance or aggregation from unrelated adults as a driver of chick aggregation formations in gentoo penguins (cf. African penguins [16]; chinstrap penguins [10]; king penguins [9]). However, the study of predator attendance was limited, as photographs were not taken frequently enough to consistently detect predation events or abundance (n = 8). Therefore, we cannot reliably determine whether predation is an additional driver of this behavior in gentoos. Likewise, gentoo chicks are particularly mobile when compared to conspecifics, so time-lapse images taken at a frequency of every hour, as is the case with this study, are limited in their ability to assess aggregation behavior when chicks move outside the boundaries of the sub-colony.

In addition, the ratio of adults to chicks did not appear to influence our measures of chick aggregations, although we did find evidence that more chicks aggregate and more aggregations occur across the species’ range when fewer chicks are guarded by their parents (Table 1). However, the relationship between aggregations and the ratio of guarded to unguarded chicks likely suggests that chicks are aggregating more when adults are absent and thermoregulation is consequently more difficult, rather than because of aggressive advances from adults. Directly quantifying aggression events would be an obvious next step to confirm this finding. Nevertheless, because adult to chick ratio has been used to determine significant trends in this behavior in the past [10], and we did not discover a relationship between this variable and aggregation measures, we believe that our results regarding unrelated adult aggression are likely accurate, even without direct observation.

We provide strong evidence that chick aggregations form more often and are larger in size at our northern-most colony on the island of South Georgia. The difference in aggregating behavior between South Georgia and our three Antarctic Peninsula colonies provides evidence that this behavior, although apparent in all four colonies, does not appear to be generalizable across the range of the species. Instead, we show that colonies exhibit distinct differences in their aggregating behavior, which is likely due to differences in environmental conditions present and may be exaggerated by the difference in the timing of the post-guard phase. Studying multiple sites within the species range is the first step in gaining a better understanding of the range of aggregation behaviors at a species level, demonstrating how colonies are related in their behaviors, and whether or not this behavior is generalizable. Single colony studies, while useful, may lack this breadth of understanding. The significant differences in behaviors between colonies, as revealed by this study, demonstrate the necessity to examine colonies at multiple locations before generalizing results, which otherwise may be oversimplified when determining the drivers of observed behaviors.

Our results highlight the utility of time-lapse cameras to remotely monitor animal behavior using scan sampling methods, without the need for *in situ* data collection [30]. The method allows for accurate measurement of the timing of behaviors and assures the standardization of sampling across multiple locations at the same time, while also limiting observer differences. We provide evidence of the strength of cameras as wide-scale behavioral sensors; however, the inability to routinely detect predation or events of adult aggression using time-lapse imagery also suggests some important limitations. Given the facility to study multiple colonies simultaneously, we were able to better understand how behaviors differ between colony locations in the same species. Future studies should evaluate multiple colonies before generalizing behaviors based on one location and past studies may need to be re-evaluated to determine whether chick aggregation and other behaviors are in fact exhibited species-wide.

## Supporting Information

S1 Table Complete data set of gentoo (*Pygoscelis* papua) chick aggregations and environmental variables at four sites. (CSV)

S1 Fig Images from the first day of the post-guard period in gentoo penguins (*Pygoscelis papua*) during the 2012–2013 breeding season at each of our four study sites: A) Maiviken, B) Georges Point, C) Port Lockroy, and D) Petermann Island. (TIFF)
